# Understanding constraints on integrated care for people with HIV and multimorbid cardiovascular conditions: an application of the Theoretical Domains Framework

**DOI:** 10.1186/s43058-021-00114-z

**Published:** 2021-02-12

**Authors:** Julie Schexnayder, Chris T. Longenecker, Charles Muiruri, Hayden B. Bosworth, Daniel Gebhardt, Sarah E. Gonzales, Jan E. Hanson, Corrilynn O. Hileman, Nwora Lance Okeke, Isabelle P. Sico, Rajesh Vedanthan, Allison R. Webel

**Affiliations:** 1 Frances Payne Bolton School of Nursing, Case Western Reserve University, 10900 Euclid Ave, Cleveland, OH 44106-7343 USA; 2 Case Western Reserve University School of Medicine, Cleveland, OH USA; 3 University Hospitals Harrington Heart & Vascular Institute, Cleveland, OH USA; 4 Duke University School of Medicine, Durham, NC USA; 5 MetroHealth Medical Center, Cleveland, OH USA; 6 New York University Grossman School of Medicine, New York, NY USA

**Keywords:** Theoretical Domains Framework, Cardiovascular disease, HIV, Integrated care models

## Abstract

**Background:**

People with HIV (PWH) experience increased cardiovascular disease (CVD) risk. Many PWH in the USA receive their primary medical care from infectious disease specialists in HIV clinics. HIV care teams may not be fully prepared to provide evidence-based CVD care. We sought to describe local context for HIV clinics participating in an NIH-funded implementation trial and to identify facilitators and barriers to integrated CVD preventive care for PWH.

**Methods:**

Data were collected in semi-structured interviews and focus groups with PWH and multidisciplinary healthcare providers at three academic medical centers. We used template analysis to identify barriers and facilitators of CVD preventive care in three HIV specialty clinics using the Theoretical Domains Framework (TDF).

**Results:**

Six focus groups were conducted with 37 PWH. Individual interviews were conducted with 34 healthcare providers and 14 PWH. Major themes were captured in seven TDF domains. Within those themes, we identified nine facilitators and 11 barriers to CVD preventive care. Knowledge gaps contributed to inaccurate CVD risk perceptions and ineffective self-management practices in PWH. Exclusive prioritization of HIV over CVD-related conditions was common in PWH and their providers. HIV care providers assumed inconsistent roles in CVD prevention, including for PWH with primary care providers. HIV providers were knowledgeable of HIV-related CVD risks and co-located health resources were consistently available to support PWH with limited resources in health behavior change. However, infrequent medical visits, perceptions of CVD prevention as a primary care service, and multiple co-location of support programs introduced local challenges to engaging in CVD preventive care.

**Conclusions:**

Barriers to screening and treatment of cardiovascular conditions are common in HIV care settings and highlight a need for greater primary care integration. Improving long-term cardiovascular outcomes of PWH will likely require multi-level interventions supporting HIV providers to expand their scope of practice, addressing patient preferences for co-located CVD preventive care, changing clinic cultures that focus only on HIV to the exclusion of non-AIDS multimorbidity, and managing constraints associated with multiple services co-location.

**Trial registration:**

ClinicalTrials.gov, NCT03643705

**Supplementary Information:**

The online version contains supplementary material available at 10.1186/s43058-021-00114-z.

Contributions to the literature

- This study adds to the HIV literature by providing a consensus-generated list of barriers and facilitators to CVD preventive care in HIV specialty care settings.
- We present management of non-AIDS multimorbidity as a unique challenge for traditional integrated HIV care models and identify clinic characteristics that may hinder collaborative care approaches to expanding CVD prevention services.
- Our qualitative findings suggest that the plurality of co-located services in HIV care settings is an important contextual factor influencing the prevention and treatment of CVD.

## Introduction

Supporting the health of people with HIV (PWH) requires prevention of non-AIDS comorbidities such as cardiovascular disease (CVD). PWH have a high prevalence of traditional CVD risk factors [1–3] and experience a twofold increased risk of CVD compared to those without HIV [4]. Their excess CVD risk results from a combination of HIV-specific factors and traditional risk factors. For the latter, evidence-informed lifestyle modifications and targeted therapies can significantly reduce CVD risk [5].

Hypertension and hyperlipidemia are CVD risk factors that impose similar chronic care needs as HIV [6], yet PWH who can successfully control their HIV often fail to receive and enact recommended therapies for prevention and control of these conditions [7–9]. Understanding barriers to CVD preventive care from the perspective of both PWH and their HIV care providers is necessary to improve CVD-related outcomes in this population.

In this paper, we report findings from a formative evaluation of CVD preventive care at study sites participating in the *Nurse-led Intervention to Extend the HIV Treatment Cascade for Cardiovascular Disease Prevention (EXTRA-CVD)* trial (NCT03643705)*.* EXTRA-CVD is an implementation trial of a multicomponent, nurse-led intervention to control hypertension, and hypercholesterolemia in individuals with well-controlled HIV infection [10]. The purpose of the formative evaluation was to identify common facilitators and barriers to CVD preventive care in participating study sites.

## Methods

To ensure quality of reporting, we adhered to the Consolidated Criteria for Reporting Qualitative Research (COREQ) (see Additional file 1) [11].

### Conceptual approach: the Theoretical Domains Framework

We used the Theoretical Domains Framework to better understand local clinic context at participating EXTRA-CVD sites, with a prespecified focus on facilitators and barriers to CVD preventive care in these settings. The TDF offers an ecological lens in which to consider multi-level influences on behavior change. It distills 84 theoretical constructs into 14 domains, and provides a validated taxonomy of implementation determinants that are applicable to health professional and patient behaviors [12].

### Study setting and participants

Participants included adults living with HIV and healthcare providers at three federally funded academic medical centers in Durham, NC (Duke Health), and Cleveland, OH (University Hospitals and MetroHealth Medical Center). Participants were recruited by purposive sampling from the HIV clinics in which they received care or worked. Individuals were approached to participate by telephone or email. PWH were eligible to participate if they were 18 years or older, received care at a participating medical centers’ HIV clinic, had a recent HIV viral load < 200 copies/ml in the prior 12 months, had documented hypertension [13] and hypercholesterolemia (a non-HDL cholesterol > 130 mg/dL) [14], or were on antihypertensive or cholesterol-lowering medication.

We sought multidisciplinary healthcare providers by recruiting physicians, nurses, social workers, and other support staff. In addition to HIV specialists, we included primary care providers who care for PWH but practice outside of the HIV clinics. This decision was driven by concern for differential CVD preventive care practices by specialty type [8], and because the HIV clinics participating in the EXTRA-CVD study differed in their provision of primary care services.

### Data collection

Semi-structured interview guides differed based on interviewee role (see Additional file 2). Interview questions were designed to elicit perceptions of CVD risk in PWH, and barriers and facilitators to high-quality CVD care. Interviews and focus groups occurred between October 2018 and January 2019. Interviewers were female team members, including a trained clinical research coordinator, a student with doctoral-level training in qualitative research methods, and a principal investigator with expertise in mixed methods research. Interviewers had no pre-existing relationships with study participants. Focus groups were held in person and lasted approximately 60 min. The number of participants varied by focus group (range, 4–9 PWH per focus group). To address challenges in focus group recruitment at Duke Health, PWH who declined to participate in focus groups were offered the option of completing a single on-campus or telephone-based interview. All healthcare providers were offered their choice of a single on-campus or telephone-based interview. Interviews lasted approximately 30 min.

All participants completed a demographics survey. PWH additionally completed the Health Beliefs for Cardiovascular Disease scale, a Likert-type questionnaire that was previously developed for use in non-HIV-infected populations [15]. The scale measures perceived CVD risk along two dimensions: perceived CVD susceptibility (5 items) and severity (5 items). Scores for each subscale range from 5 to 20. Two additional subscales capture perceived barriers and benefits to engaging in CVD prevention. Scores range from 6 to 24 for the perceived benefits subscale (6 items) and from 9 to 36 for the perceived barriers subscale (9 items). For all subscales, higher values indicate more of these perceptions [15].

Focus groups and interviews were audio recorded and professionally transcribed. Study procedures were approved by the University Hospitals (UH) Cleveland Medical Center IRB with reliant review agreements at MetroHealth and Duke Health. All participants provided written consent to participate.

### Data analysis

Transcripts were managed using Dedoose Version 8.0.35 [16]. Data were analyzed using a highly structured form of thematic analysis [17]. Our preliminary codebook consisted of the 14 domains of the TDF. We coded 10 percent of transcripts with the preliminary codebook, and iteratively revised the code descriptions in group discussions. The finalized codebook was applied to all transcripts. Each transcript was independently coded by two study team members (CM, IS, JH, and JS). Coding disagreements were resolved through consensus. For the PWH dataset, we elected to code data at the individual level to address differences in data collection methods (i.e., interviews and focus groups).

Selection of themes occurred in three stages. Data from PWH and healthcare providers were analyzed separately. In the first stage, study team members (CM, JH, JS, IS, and AW) independently completed analysis worksheets to identify themes emerging in coded text. Themes were discussed over 10 meetings and were retained for further analysis when a theme was present in multiple analysts’ worksheets. Next, two team members (JS and AW) reviewed each theme’s relevance to CVD prevention and determined the best fitting theoretical domain for themes emerging from two or more domains. In stage three, transcripts were re-reviewed (by JS and AW) to verify theme presence, fit, and depth of content. At this stage we considered participants’ scores for the Health Beliefs for Cardiovascular Disease Instrument [15] to inform analysis of the PWH dataset and re-examined focus group and interview transcripts to assess for differences in thematic content. Final themes were present and achieved saturation at two or more study sites. Feedback on final themes was obtained from healthcare providers and PWH that participated in the EXTRA-CVD intervention design team as a proxy for member checking.

## Results

In total, 85 individuals completed an interview or focus group. Thirty-four healthcare providers completed interviews. Healthcare providers were majority female (67.7%) and White (73.5%). The most commonly represented healthcare providers were physicians (44.1%), registered nurses (23.5%), and social workers (17.6%). Most healthcare providers self-identified as HIV specialists (78.8%) and had an average of 14.5 years of experience providing care to PWH (Table 1).

**Table 1:** Participant demographics (*n = 85*)

Characteristic	People with HIV (*n = 51*)	Healthcare providers (*n = 34*)
	Number (%)	Number (%)
Age, mean (SD)^a^	57.0 (7.8)	47.4 (11.4)
Sex		
Female	17 (33.3)	23 (67.6)
Male	34 (66.7)	11 (32.4)
Race		
Asian	0 (0)	4 (11.8)
Black/African American	31 (60.8)	4 (11.8)
Hispanic^b^	7 (13.7)	0 (0)
White/Caucasian	9 (17.6)	25 (73.5)
Multiple/other	4 (7.8)	1 (2.9)
Highest completed education		
Less than high school degree	6 (11.8)	0 (0)
High school or GED	12 (23.5)	0 (0)
Some college	23 (45.1)	4 (11.8)
Bachelor’s degree	4 (7.8)	7 (20.6)
Master’s degree	6 (11.8)	7 (20.6)
Doctorate degree	0 (0)	16 (47.1)
Professional role		
Licensed social worker	--	6 (17.6)
Physician	--	15 (44.1)
Registered nurse	--	8 (23.5)
Physician	--	15 (44.1)
Other	--	5 (14.7)
Employment status		
Currently employed	15 (29.4)	--
Temporarily unemployed	3 (5.9)	--
Retired	9 (17.6)	--
Disabled	22 (43.1)	--
Other	2 (3.9)	--
Monthly income (USD), median (IQR)	983 (1100)^c^	--
Insurance status		
Public	37 (72.5)	--
Private	11 (21.6)	--
Public and private	1 (2.0)	--
Other	1 (2.0)	--
Uninsured	1 (2.0)	--
Time since HIV diagnosis (years), mean (SD)	18.7 (6.4)^d^	--
Mean (SD) years professional HIV experience	--	14.5 (9.6)
Perceived cardiovascular risk		
Perceived CVD susceptibility, mean (SD)^e^	10.8 (3.9)	--
Perceived CVD severity, mean (SD)^f^	9.6 (3.5)	--
Perceived benefits to CVD prevention, mean (SD)^g^	9.5 (5.0)	--
Perceived barriers to CVD prevention, mean (SD)^h^	17.0 (4.9)	--
Study site		
Duke Health	18 (35.2)	12 (35.3)
MetroHealth	17 (33.2)	12 (35.3)
University Hospitals Cleveland	16 (31.37)	10 (29.4)

^a^Self-reported age available for people with HIV (*n = 49*) and healthcare providers (*n = 29*)

^b^Individuals reporting Hispanic ethnicity were counted as Hispanic only. All other categories reflect non-Hispanic status

^c^Self-reported income available for people with HIV (*n = 48*)

^d^Time since HIV diagnosis available for 48 people with HIV

^e^Scores for this subscale range from 5 to 20; higher values indicate more of these perceptions; (*n = 47*)

^f^Scores for this subscale range from 5 to 20; higher values indicate more of these perceptions; (*n = 44*)

^g^Scores from this subscale range from 6 to 24; higher values indicate more of these perceptions; *(n = 47*)

^h^Scores from this subscale range from 9 to 36; higher values indicate more of these perceptions; (*n = 47*)

Six focus groups were conducted with 37 PWH. An additional 14 PWH who received care at Duke Health completed interviews. PWH were majority male (66.7%) and Black/African American (60.8%). Approximately 65% of PWH had completed at least some college and reported a median monthly income of $983. The majority of PWH reported public health insurance (72.5%). Time since HIV diagnosis ranged from 6 to 31 years. A greater proportion of focus group participants reported their employment status as unemployed on disability (54.1%) as compared to interview participants (14.3%), but they were otherwise demographically similar in age, gender, income, insurance status, educational attainment, and time since HIV diagnosis. Perceived CVD susceptibility [median (IQR) = 12 (7–14)] and severity scores [median (IQR) = 9 (7–12)] among PWH were consistent with low perceived CVD risk. On average, PWH perceived low barriers to engaging in CVD prevention [median (IQR) = 16 (13–21)], but also low benefits [median (IQR) = 7 (6–11)].

Qualitative results are presented by theoretical domain. Seven theoretical domains explained factors influencing CVD prevention in HIV care settings (Table 2). Descriptions of explanatory themes are provided for each domain, including identification of facilitators and barriers associated with those themes. The *Knowledge* domain represented the greatest number of themes (*n* = 4), followed by *Environmental Context and Resources* (*n* = 3); *Beliefs about Capabilities* (*n* = 2); and *Memory, Attention, and Decision Processes* (*n* = 2). The *Behavioral Regulation*, *Beliefs about Consequences*, and *Social and Professional Roles domains* each represented a single theme. In total, eight themes emerged in both HIV care team member and PWH transcripts.

**Table 2:** Explanatory themes by theoretical domain

TDF domain	Domain description	Theme	Groups endorsing theme^1^			Facilitator^2^	Barrier^2^
			Duke	Metro	UH		
Knowledge	Discussions of CVD risk including scientific rationale for risk factors and prevention, procedural knowledge of CVD risk identification and management (including self-management), and discussion of associated knowledge sources	HIV as a risk factor for CVD	1, 2	1, 2	1, 2	X	
		Multiple sources of CVD-related information	2		2	X	
		Translating knowledge of discrete diagnoses and behaviors into composite CVD risk	2^§^	1, 2	1		X
		CVD-related medications and their mechanisms of action	1	1, 2	1, 2		X
Environmental Context and Resources	Discussion of environmental stressors, organizational culture or climate, salient critical events. or other incidents influencing goals or processes related to CVD	Co-located services	1	1	1	X—access to resources	X—bottlenecks in clinic flow
		Social determinants of health	1, 2	1	1, 2	X—health insurance^3^	X—transportation^4^
		Frequency of care to manage non-HIV conditions	1	1	1		X
Beliefs about Capabilities	Reflections on self-confidence, perceived competence, self-efficacy, self-esteem, perceived behavioral control, empowerment, and/or professional confidence in identifying and managing CVD	CVD complexity	1	1	1	X	
		Locus of control in preventing and managing CVD		2	2		X
Memory, Attention, and Decision Processes	Description of what CVD-related factors individuals choose what to pay attention to and what they ignore, including discussions of competing demands, cognitive overload, burnout. or decisional aids to support CVD-related behaviors	Tools to aid CVD prevention	1, 2	1, 2	2	X—tools for CVD risk estimation^4^, medication reminders3	X—tools to motivate lifestyle modifications^3^
		HIV is our priority	1, 2^§^	1, 2	1, 2		X
Behavioral Regulation	Reflections on the use of objective measurements to make changes in CVD-related risks or behaviors	Self-monitoring practices	2	2	2	X—the value of self-monitoring	X—symptom guided self-management
Beliefs about Consequences	Discussion of outcomes, anticipated regrets. or other consequences associated with CVD (risk or disease) or CVD- related behaviors	Medication burden; subtheme: past experiences with CVD medication side effects	1, 2	1, 2	1, 2		
Social and Professional Roles	Discussions of who holds responsibility for CVD-related tasks/behaviors	Safety net primary care	1, 2	1, 2	1, 2	X-HIV providers as trusted sources of medical advice	
							X-Decisions to manage CVD for PWH
							X-HIV provider messaging on CVD risk

^1^Participant groups: 1 = healthcare providers, 2 = people with HIV (PWH)

^2^Subthemes are presented for explanatory themes representing a combination of facilitators and barriers

^3^Subtheme identified in PWH dataset only

^4^Subtheme identified in healthcare provider dataset only

^§^Theme present in PWH interviews only

### Knowledge

Two facilitators and two barriers were described in relation to the *Knowledge* domain. Healthcare providers and PWH identified HIV and certain HIV medications as independent risk factors for CVD (facilitator). Although PWH described biobehavioral CVD risk factors, they experienced challenges in estimating their overall heart health in relation to their personal diagnoses and health behaviors (barrier). This knowledge-application discrepancy was echoed by healthcare providers,

“I think a lot of patients don’t connect that diabetes can lead to heart disease. So, making that awareness and saying, ‘In diabetes, we’re not just treating diabetes, we’re preventing other things.’” (Physician, 24 years HIV experience)

Inadequate knowledge of the medications PWH used to treat their hypertension and hyperlipidemia was evident in PWH descriptions of their prescribed regimens (barrier),

“Well, I asked my doctor. I said, I’m 140lbs. They give the same medicine to somebody 300 lbs. That don’t make sense to me. And that’s why I’m ask, how do you calculate how everything else is done? They do your – with weight. I can’t see why I need the same amount, especially the high blood pressure medicine.” (PWH, 9 years with HIV)

For some, the knowledge deficit extended to understanding of normal blood pressure or lipids values (barrier), particularly when those values occurred in the context of recent medication use. Healthcare providers described efforts to remediate those knowledge deficits,

“Sometimes they ask, ‘why do I have to be on all of these medicines, and can I be on less, can I stop taking them because my blood pressure is good’ and that will open up a dialogue. Well, it’s probably good because you’re taking the medicines.” (Physician, 15 years HIV experience).

Despite their knowledge deficits, PWH identified wide-ranging sources of information on CVD and CVD risk management (facilitator). Multiple information sources allowed PWH to validate the guidance they received from members of their HIV care team. PWH expressed confidence in their healthcare team’s recommendations when the recommendations were consistent with other information sources. However, approaches to managing conflicting information differed, with some PWH choosing to self-adjust therapies:

“One of the nurses [from the insurance company] said I should talk with my doctor because it seems that the dosage for my cholesterol medication was too high. I emailed my doctor and she said you’re fine. So, I’m hearing this from my doctor and then I’m hearing this from the nurse from the insurance company. So, I’m like, okay. I’m going to do in the middle. And I’m going to take them every other day, okay.” (PWH, 9 years with HIV)

### Environmental context and resources

Two facilitators and three barriers were described in relation to the *Environmental Context and Resources* domain. Healthcare providers identified service co-location as a distinguishing feature of HIV clinics, and an important source of PWH self-management support (facilitator). At least one co-located, cardiovascular health program was offered at each clinic (i.e., tobacco cessation, exercise classes, dietician, and/or cardiovascular specialty care). Co-located services were also described by healthcare providers as a source of bottlenecks in clinic flow, limiting their time to address patients’ CVD risk factors during routine encounters (barrier):

“We have such great services, more than any other clinic in the hospital because of the funding that’s available to HIV… So, because of all of these services we do not have space and we do not have time. It’s something that we struggle with all the time. It creates tension between staff members, this pull on patients and their time. We could probably inundate a patient with at least six staff members every time they come into clinic because of all the things.” (Licensed Social Worker, 14 years HIV experience).

In addition to time constraints, HIV physicians also identified visit frequency as a barrier to managing CVD in HIV clinics.

“Sometimes I will refer patients to primary care providers because if I’m seeing a patient, if their HIV’s well-controlled, I’m seeing them once every six months or sometimes once a year, and it’s not frequent enough to be able to check their blood pressure or their plans for exercise and diet, etc.” (Physician, 12 years HIV experience)

Finally, the influence of social determinants of health on PWH’s ability to self-manage CVD risk was discussed. For PWH, health insurance was an important facilitator of CVD care and gained PWH access to additional health resources outside of clinic. Benefits included gym memberships, medication safety programs, and nurse-run telephone hotlines. Healthcare providers focused on unstable transportation access for many PWH and its impact on medical visit attendance (barrier).

### Beliefs about capabilities

One facilitator and one barrier were described in relation to the *Beliefs about Capabilities* domain. HIV specialists already conducted routine screening and counseling for common CVD risk factors, namely hyperlipidemia, hypertension, and smoking (facilitator). Medical complexity was a critical factor influencing HIV specialists’ comfort with managing CVD in HIV care settings. When medical cases were complex, HIV specialists differed in their comfort treating PWH without specialty guidance, but in general, were less comfortable managing complex CVD-related medication regimens as compared to primary care providers,

“Maybe I can…hold off on referring, depending on the severity of course, and the complexity, …but it’s one thing to correct cholesterol with diet. It’s an entirely other thing when you’re trying to correct really complicated dyslipidemias, for example, which can involve three or four subclasses of lipid.” (Physician, 20 years HIV experience)

PWH also described complexity related to the large number of factors influencing CVD risk and their varying locus of control in managing those factors. Whereas PWH believed they could reduce one or more of their CVD risk factors, they also acknowledged limits on their ability to eliminate CVD risks due to HIV (barrier),

“Now, if I know how HIV medicine reacts with my cholesterol, then what I have to do is take my [HIV] medicine, but I can also cut back on fatty foods and stuff like that, work on the other risk factors. I can’t work on the HIV other than taking my medicine, but I can work cholesterol and stuff with exercise, diet, etc.” (PWH, 6 years with HIV)

### Memory, attention, and decision processes

One facilitator and two barriers were described in relation to the *Memory, Attention, and Decision Processes* domain. Healthcare providers and PWH extolled the benefits of support tools. For healthcare providers, CVD risk calculators were indispensable tools for counseling and medical decision-making (facilitator). Ease of access to a CVD risk calculator was dependent on whether or not the tool was directly accessible in the electronic medical record,

“I think the dot CVD phrase, is a huge time saver. It’s just few keystrokes and boom, all the data are there. That was a really helpful development in our clinic.” (Physician, 24 years HIV experience)

PWH described various treatment adherence aids, ranging from pill boxes to electronic reminders and trackers. Multiple PWH had tools which worked for them to establish adherent HIV medication habits and felt those experiences promoted adherence to other (CVD) medications (facilitator). Whereas tools to support medication adherence were common, tools to motivate and sustain other lifestyle modifications were identified as unmet needs (barrier).

Although healthcare providers valued tools to assist with CVD screening and treatment, there was strong agreement that HIV was the priority focus during patient encounters and that CVD increased in importance as individuals sustained HIV viral load suppression. PWH also prioritized HIV self-management over other health conditions, often due to perceived severity of HIV disease,

“To me, that’s the ultimate, my HIV. But then my mental health comes in second. And my physical heart health, yeah, I take care of it when I can, but I haven’t entirely quit smoking. The amount of exercise, it depends on where I leave my remote control. I can’t be bothered. Which is not a good thing and shame on me.” (PWH, 22 years with HIV)

### Behavioral regulation

One facilitator and one barrier were described in the *Behavioral Regulation* domain. Both related to PWH self-management practices and emerged exclusively in PWH transcripts. PWH valued and engaged in a number of CVD-related self-management practices (facilitator) but differed in their motivations to do so. Those who independently engaged in self-monitoring of CVD risk factors expressed an increased understanding of how their behaviors influenced their health,

“It can take time to get used to monitoring it. But the benefit behind doing all of that is that now I see that the things that I do make a big difference in my blood pressure now compared to then, it gives you a little bit more enthusiasm to stay on it.” (PWH, 18 years with HIV)

Other PWH were motivated by physical symptoms or abnormal values. This group focused on hypertension self-management, with symptoms or elevated blood pressure values triggering time limited attention to medication adherence and blood pressure self-monitoring (barrier). Focus groups elicited additional depth on how some participants learned to respond to elevated blood pressure readings.

PWH 1, 25 years with HIV: “So, I have done it a couple times. But I do the same thing, I’ll go to CVS, sit down, and take it, and then I wait, and then I take it one more time.

Moderator: Okay, and so that waiting that you guys do, did someone tell you to do that, or did you just figure it out?

PWH 2, 18 years with HIV: They kind of told us – usually, if I go to the hospital that’s the first thing they do when you come in. So, if you’re around waiting long, you’re still probably hyped-up from getting here and, you know.

PWH 3, 24 years with HIV: Mm-hmm, and it always seems high.

PWH 2: And it's higher, and then she said, “I'll take it later,” a lot of times she'll take it 15 minutes later, and it'll be lower; so just from experience you find that that's the case. And then, sometimes they'll take it two or three times just to make sure.

PWH 1: It was the same thing for me, yeah. I just sort of understood that if you waited a little bit, and calmed down, and don’t take it when you’re running…”

### Beliefs about consequences

One barrier was described in relation to the *Beliefs about Consequences* domain and was congruent in both the HIV care team and PWH transcripts. In it, the consequences were associated with medication therapies rather than CVD itself. PWH associated treatment of CVD with increased medication burden. Many were adherent to their medication regimens but acknowledged a preference for taking fewer medications. Some wanted to know they had exhausted options for lifestyle modifications before agreeing to new CVD-related prescriptions. For others, medication burden influenced their decisions to adhere to prescribed regimens and their willingness to present for CVD-related medical care,

“One of my biggest deterrents to seeing any physician –– my doctor's been pushing me toward visits with a heart doctor—it’s like my concern is I don't want to take any more medications than I absolutely, positively have to. So often you go to a doctor, and you walk out of there with two or three prescriptions; and I'm like, ‘No, I don't want it, I don't want any more prescriptions!” (PWH, 18 years with HIV)

HIV care team members also recognized the impact of medication burden on their patients and described the conflicts that arise when making recommendations for additional, non-HIV-related medications,

“No one ever comes in and says, ‘I want to be on a statin.’ Blood pressure is the same thing. ‘Oh, no, no. My blood pressure at home is 120. It’s not really 180. I don’t like that. Please, Doctor, don’t give me any more medicine.’” (Physician, 13 years HIV experience)

In addition to the number of medications, side effects were a source of medication burden for PWH. PWH who, in the past, experienced side effects from CVD medications were particularly concerned about the potential side effects of new medications. Descriptions of side effects differed by drug class and ranged from minor to serious adverse effects requiring emergency care.

### Social and professional roles

One facilitator and two barriers were described in relation to the *Social and Professional Roles* domain. As specialists, HIV providers viewed their role in CVD prevention as safety net care providers and differed in their willingness to provide CVD preventive care.

“As an HIV specialist that also is, to some extent, charged with taking care of the primary care issues that patients who don’t have primary care physicians, we don’t have an infrastructure that works well to allow for excellent communication between practices.” (Physician, 20 years HIV experience)

For healthcare providers, co-management by HIV and primary care teams also introduced a need for defining professional boundaries regarding CVD management roles (barrier). CVD prevention was regarded as a primary care activity. However, because of their long-term relationships with patients and their tendency to fill gaps in their patients’ primary care when needed, HIV specialty care providers felt that they were their patients’ trusted source of CVD-related medical advice (facilitator).

Multiple PWH described that, after years of relying on their HIV clinics for their general medical care, they were directed to obtain a primary care provider. Some PWH were open to pursing primary care for their age-related conditions, whereas others preferred to continue with their current care,

“I just have an HIV doctor, and he asked me the last time I was in if I had a primary care doctor. And I said, ‘That’s you!’ He said, ‘No, it’s not me”. And I said, ‘I just have HIV, nothing else seems to be wrong with me, so you’re my primary care doctor until such time as I feel I should see one.’” (PWH, 25 years with HIV).

Focus groups, in particular, elicited content on how changing provider roles intersected with patient preferences for their HIV provider’s involvement in managing their non-HIV care.

PWH 1, 26 years with HIV: “So, for me, now I have a primary care doctor and I have an HIV doc. My HIV doctor only wants to treat my HIV, not the whole thing which is a little frustrating. My personal doctor has a good handle on HIV as well but she leaves that part to him. But he doesn’t really want to do anything else.

PWH 2, 24 years with HIV: That’s interesting because to me it has to be integrated. Mine is reversed, thank goodness. The ID doctor is much more involved in my primary care than my primary care doctor – I’ve even discussed with my HIV doctor that my primary care is only there to write prescriptions. That’s just an honest assessment.

PWH 1: My last one was. Now it’s the exact opposite.

PWH 2: That’s interesting. I hear you. That’s a bad reverse, in my opinion.”

Finally, some who relied on their HIV care teams for general medical care assumed that absent or superficial discussions of CVD meant that they were not at significant risk (barrier). This was because they trusted their HIV providers would notify them of important health risks regardless of whether their HIV providers had explicitly agreed to manage non-HIV conditions.

## Discussion

Using the TDF, we observed agreement on multiple barriers to CVD preventive care in EXTRA-CVD study sites. Our most striking finding is the need for improved care integration for PWH who have multiple chronic conditions. Integrated care is the creation of structural links between separate healthcare services, with a goal of reducing fragmentation and enabling more coordinated and continuous care [18]. In the USA, integrated HIV care is common and frequently combines case management, behavioral health care, or social support services to promote longitudinal retention in care and HIV viral suppression [19–22]. In this formative evaluation, HIV clinics offered services consistent with traditional integrated HIV care models, but the scope of primary care services available to PWH varied considerably. For those with CVD-related conditions, our findings highlight gaps in this traditional model as well as challenges to communication and follow-up when crossing multiple treatment areas.

It is presently unclear if a particular integrated care model is superior for CVD prevention treatment in PWH. In low- and middle-income countries, efforts to vertically integrate non-AIDS comorbidity and HIV care are common but these programs are frequently limited to a single health condition [23, 24]. In the case of CVD prevention, multiple health conditions (diabetes, hypertension, hyperlipidemia, obesity, tobacco use disorder) must be effectively screened and managed [25]. In the USA, there are limited evaluations of HIV clinics that integrate multiple CVD preventive services [26]. The few examples [27–29] do not address the full cache of conditions needed for comprehensive CVD prevention and present mixed findings when comparing outcomes to shared care or consolidated care models. Additional study is needed to determine how successful integrated care models can be expanded to address all of the CVD prevention needs of PWH [24]. Pending additional research, our findings support integrated HIV and CVD services that can be individualized to patients’ preferences and be sensitive to care coordination needs that are likely to differ when health services are provided by multiple care teams [30].

Tailoring integrated care models and providing support during implementation may be necessary for integration to occur outside of research settings [31]. From an implementation perspective, EXTRA-CVD shares features with the Collaborative Care Model, an approach to primary care mental health integration wherein a primary care provider provides behavioral health services to patients with support from a care manager and consultation from a psychiatrist who provides treatment recommendations for patients who are not achieving clinical goals [32]. In the EXTRA-CVD model, the HIV specialist may retain responsibility for managing CVD-related medication therapies with support provided through the intervention’s four evidence-based components: co-located care coordination and medication adherence support, medication protocols, home BP monitoring, and electronic health records support tools [10].

Our findings identified multiple contextual factors relevant to EXTRA-CVD implementation. First is the reluctance of multiple PWH to engage with cardiovascular specialists and primary care providers for CVD preventive care. This may be explained in part by our findings that PWH experience differential trust in their HIV providers’ ability to manage their complex health needs. Negative past experiences with and current perceptions of HIV stigmatization by healthcare providers have been cited as potential causes [33], but neither were strongly present in this evaluation. Rather, avoidance of additional medications served as an important determinant, reinforcing the prevalence of multimorbidity for PWH [34], and the primacy of managing medication burden in a population that often experiences drug polypharmacy [35]. These findings demonstrate patient acceptability for the physical and institutional structures of EXTRA-CVD—an important facilitator of non-AIDS comorbidity and HIV care integration in other studies [36].

Whereas many PWH preferred HIV-based care, HIV specialists differed in the CVD-related responsibilities they were willing to accept. Garnering buy-in from HIV specialists will be essential to implementation given their central prescribing role. Nearly all HIV specialists described CVD risk factor screening practices that were consistent with HIV care guidelines developed by infectious disease societies [37]. Yet treatment of common CVD risk factors was often described as a primary care activity for others to manage. This finding is consistent with other specialists’ reluctance to expand their CVD prevention practices across specialty domains [38] and presents a common implementation challenge for programs requiring deviation from traditional staff roles. Concerns about practice independence and inability to practice to full scope have been shown to impede implementation [39–41] and may be of significant concern to infectious disease specialists who must balance expanding primary care roles with the demands of maintaining a specialty knowledge base. Although stakeholder engagement strategies have been incorporated into the EXTRA-CVD design [42], provider readiness is likely to be an important moderator of EXTRA-CVD fidelity and requires further study. The uptake and impact of EXTRA-CVD may differ substantially for PWH whose CVD-related conditions are co-managed by primary care providers.

In either care model, care coordination remains an essential component of the EXTRA-CVD intervention with information monitoring and sharing as key processes. The teamwork factors influencing care coordination are incompletely characterized, and it is important to consider that some aspects of care coordination may be fundamentally different when delivered across versus within care teams [43]. Findings of nebulous provider roles in shared care and suboptimal communication described by some HIV providers in our study are contextual factors that may increase the complexity of care coordination. Notably, the electric medical records features described in this study as most useful for CVD prevention had minimal perceived utility for communicating care needs to other members of the healthcare team. Understanding how to maximize the communication potential of electronic health records for care coordination remains a critical research priority [44]. Therefore, implementation support may be needed to select care coordinators with the appropriate communication skills [45] and to promote effective communication and collaboration between the EXTRA-CVD care coordinator and other members of the care team [45–47].

Care coordination efforts may also be hindered by the prioritization of HIV by PWH and their HIV care teams to the detriment or exclusion of comorbidity care. PWH perceived their CVD-related conditions as less serious than HIV, and selectively tended to prioritize HIV self-management in response. This practice is not unique to HIV. In their systematic review, Koch et al. identified disease prioritization as a common experience in patients managing multiple chronic conditions [48]. In our study, PWH may have assigned lower priority to their CVD risks because they had not experienced significant CVD complications and because they experienced significant knowledge gaps regarding CVD prevention therapies. These findings can contribute to the development of patient education and adherence support activities delivered by the EXTRA-CVD care coordinators. However, patients’ focus on HIV may be further reinforced by their HIV care teams—on whom the EXTRA-CVD care coordinator may rely for patient referrals. Implementation support may be necessary to promote visibility of the care coordinator as member of the HIV care team [46] and reframe clinic perceptions of CVD prevention as an important component of integrated HIV care.

Finally, multiple services co-location appears to be an important factor that is unique to specialty care settings. Co-location was distinguished as an important facilitator and barrier to CVD prevention and likely to influence opportunities for providers and EXTRA-CVD care coordinators to engage in preventive care. Each of the study sites offered multiple co-located services for PWH. This is likely related to their current and historical funding mechanisms that incentivize integrated service models [49]. The presence of co-located services was widely viewed by participants as beneficial to increasing patient access to care; however, it only partially addressed patients’ transportation and other access barriers. This may be because there is a limit to the number of services an individual could reasonably access during a single clinic visit. The plurality of service co-location may be a unique feature of HIV care settings, but the time and space constraints and disease prioritization that occur with co-located care are not disease specific [30]. Given the range of chronic conditions for which PWH have increased susceptibility, the boundaries of integrated care models for optimizing health outcomes in specialty settings must be explored.

This evaluation highlighted several barriers to CVD preventive care that further informed intervention design. Findings were summarized and presented to the EXTRA-CVD intervention development team as part of the human-centered design process [42]. Key points were the following: (1) HIV prioritization occurs commonly in PWH and their HIV care teams and may create impediments to engaging PWH in CVD preventive care. (2) Effective integrated care models will likely require the flexibility to accommodate patient preferences for accessing CVD prevention in HIV specialty clinics, while improving non-HIV care coordination when PWH are co-managed with other clinical care teams. (3) Given the plurality of co-located services in these settings, alternatives to “add-on” care during routine HIV encounters may be needed to avoid exhaustion of the clinics’ physical resources.

By using the TDF, we were able to obtain a granular understanding of the facilitators and barriers to CVD preventive care in HIV care settings. While this enabled a comprehensive evaluation, there were notable challenges. Interview content often overlapped TDF domains and limited guidance is available for managing such overlap [50]. This increased the workload of resolving coding disagreements, which can be common with TDF use [51]. Integrating findings from PWH and healthcare provider interviews made it possible to prioritize barriers for intervention based on patient and provider consensus, but the TDF does not provide for specification of barriers that are most important to behavior change.

Our study has several limitations. First, participants were recruited from three large, academic medical centers and results may not be generalizable to all HIV care settings. Second, although we systematically applied the TDF to our data, the TDF was not used to develop the semi-structured interview guides. Thus, it is possible that information on some TDF constructs that are relevant to CVD preventive care was not elicited. However, the TDF has been used successfully in secondary qualitative data analyses [52], and a study team member (JS) mapped interview guide concepts to TDF domains a priori to ensure the framework was appropriate for use in HIV care team and PWH datasets. Therefore, the TDF was well suited to our intersecting analysis of HIV care team and PWH data. Finally, we experienced differential acceptability of focus group data collection methods by study sites. Although we identified differences in employment status among participants at Duke Health, other factors are likely influence acceptability of focus groups in this population, including prior experience with group activities that involve HIV disclosure. Notably, focus groups at all study sites included discussion of HIV support group participation, but such content was rare in interviews. Allowing for individual interviews and coding data at the individual level ensured adequate representation of PWH receiving care at Duke Health, but consequently limited exploration of how PWH described their CVD-related care with and in relation to other PWH with similar CVD risk factors. Restriction of the PWH dataset to focus group data did not result in changes to the final set of reported themes but failed to capture that two of the barriers to CVD prevention identified in this study also affected PWH receiving care at Duke Health (Table 2). Despite these limitations, we attained saturation for a relatively large number of cross-site themes, and successfully identified barriers to CVD preventive care that can be addressed to improve the care of PWH.

## Conclusions

When applied systematically, the TDF can be used to understand determinants of CVD preventive care in HIV treatment settings. We observed nine common facilitators and 11 common barriers to CVD preventive care across HIV specialty clinics in three academic medical centers using this approach. Our findings emphasize the need for strategies to improve care integration for the population of PWH with CVD-related conditions. The EXTRA-CVD intervention incorporates tailoring of strategies to mitigate barriers to CVD preventive care for PWH. Efficacy of the tailored EXTRA-CVD intervention will be assessed in an ongoing randomized, controlled trial.

## Supplementary Information

**Additional file 1:.** Consolidated Criteria for Reporting Qualitative Studies (COREQ): 32-item Checklist

**Additional file 2:.** Interview Guides

## Data Availability

The datasets used and/or analyzed during the current study are available from the corresponding author on reasonable request.
