# Supplementary material for: Understanding constraints on integrated care for people with HIV and multimorbid cardiovascular conditions: an application of the Theoretical Domains Framework
Source: Implement Sci Commun. 2021 Feb 12;2:17. doi: 10.1186/s43058-021-00114-z (PMC7881687; doi:10.1186/s43058-021-00114-z)
Supplement: Supplementary file 2 — Additional file 2:. Interview Guides [file 43058_2021_114_MOESM2_ESM.docx]

Additional File 2. Formative Evaluation Interview Guides

**Interview Guide 1: Prescribing Clinicians (MD, NP, Pharmacist, DO and PA)**

**Training Note:** Not all questions will be asked explicitly. Participants may answer the questions, or cover the topic, in the course of the focus group or one-on-one conversation.

**Introduction**

[Remind about Audio Recording and not to use real names]

Thank you for talking with us today. We are interested in learning more about your thoughts and beliefs about cardiovascular disease among your HIV+ patients. We are most interested in the patients you see in their routine, outpatient HIV care, not those in the hospital. I am going to ask you some questions about these topics to hear about your experiences and perspectives. Please know that there is no right or wrong answer. You will notice that I won’t give you a lot of feedback on your responses because I don’t want to influence your answers. You may also notice that I will jot things down on paper while you talk- this is simply a reminder to ask you a follow-up question. Finally, you are under no obligation to talk about anything that you are not comfortable discussing with me. Do you have any questions or concerns before we begin?

[Facilitator, please note the participant’s ID number in the audio recording so we can match it to the demographic information.]

***General CVD Perceptions***

1. Let’s start by you telling me about what you think the biggest health problems are for your HIV+ patients. Among these problems, where does heart disease rank?
2. Have you ever talked with your patients about heart disease? If yes, tell me about that/those conversation(s)?

**Prompts**: What prompted that discussion (e.g., did you bring it up, did the patient have abnormal blood pressure or cholesterol?) Did they ask you about their risk of developing heart disease? Did their primary care provider reach out to you?

1. How do HIV medications affect your patient’s risk of developing heart disease? How or where did you learn this?
2. Is there an intervention for your clinic to help reduce your patients’ risk of CVD? If so, how could it be improved and if not would you design it? Have you encountered any difficulties with intervention implementation? Probe: how have/would patients, other providers in the clinic and the health system be engaged in this intervention?

**Prescribing questions**

1. What helps you better manage CVD in your HIV+ patients?
2. What are the medications that you prescribe to prevent heart attacks and strokes?
3. What is your routine for monitoring cholesterol in your patients? Are there any guidelines you use to help with monitoring and managing your patient’s cardiovascular risk?
4. How often do you review current or past cholesterol results for your patients? Prompts: every visit? yearly?, rarely?
5. Vitals are typically measured at every clinical visit. Among your patients, what vitals do you mostly focus on and why?
6. How have the current guidelines about the definitions and management of high blood pressure affected your CVD management for your HIV+ patients?
7. How comfortable do you feel prescribing hypertension and cholesterol medications and managing their side effects?
8. How often do you prescribe home blood pressure monitoring? Why this frequency/ proportion of time? How do you follow up on adherence to these cardiovascular medications or monitoring? Prompts: Have your nurse call; patient calls in with numbers; check in with patient via email or EMR reminders.
9. Tell me about the conversations you have with your patients about how HIV medications affect heart disease risk?
10. Do you believe drug-drug interactions are a significant barrier to prescribing preventive medications like statins?
    - **HIV specialty providers**: If a non-HIV doctor prescribes a medication for your patients, do you know about it? How frequently do you encounter dangerous drug-drug interactions that result from others being unfamiliar with ART?
    - **PCPs (i.e. non-HIV provider)**: How knowledgeable are you about ART drug interactions? Do you avoid certain statin medications in all HIV patients for fear of drug interactions or do you check for specific interactions based on the patient’s ART?
11. Would your patients be more likely to take a preventive medication (like aspirin or statins) if their HIV doctor prescribed it compared to a non-HIV provider? Why?
12. What types of patients do you refer to a specialist for cardiovascular disease prevention? **Prompts**: very high LDL or triglycerides (do you have a threshold)? Difficult to control hypertension (do you have a threshold—i.e. not controlled on 3 meds? 5 meds?), + family history? patient preference/request?
13. How comfortable are you ordering cardiovascular testing (i.e. stress tests, echocardiograms, Holter/event monitors, coronary calcium scans)?

***Intervention Feasibility and Usability***

Finally, we are interested in designing a new initiative to help your patients reduce their risk of heart disease. However, we know in this and many clinics, there issues that can make a new initiative more or less successful. We’d like to get your opinion on these issues and as a reminder, all information you provide will be confidential and aggregated across three clinics.

*System*

1. Tell me about your clinic’s culture. What aspects of it help you do your job well and what aspects can make it harder for you? What are the strengths of your clinic?
2. Does your clinic have a hierarchy? If so, can you tell me how that affects how decisions are made in your clinic?
3. What the priorities or mission of your clinic? Have they change over time?
4. Tell me about a clinic initiative that was recently developed to help your patients improve their health behavior. What steps were taken to help make the change? Who initiated the change? Who were the people that helped drive that change and what was their role? How long did it last? What was the outcome (did it work)?
5. What factors do you think would encourage clinicians to adopt a new program to improve the heart health of patients in your clinic?

*Training*

1. What sort of education or training do you prefer for new initiatives? Do you prefer paper, verbal (conversations), digital (phone, computer) or visual (TV) training? Individual or Group? Does continuing education credit entice you to complete new trainings? Would you be interested in helping to develop a new training for your clinic on reducing heart disease in your patients?

*Design Considerations/Representations*

1. When you have a patient concern, for example an elevated temperature or a high blood pressure, how does that information get turned into action in this clinic? Can you walk me through the various steps? [consider drawing this out on white board]. How do you involve non-HIV clinicians, for example your patient’s primary care provider, in this communication cycle?
2. How often do you personally communicate with your patients outside of the clinic setting? How does this communication happen (e.g., phone, EMR, via your nurse). Does the patient usually initiate this contact or do you?

**Secondary/Follow-up Questions**

Follow-up “Probes” after significant statements are made:

[Earlier/A moment ago/when you first started speaking/when you were talking about x] you said [significant statement].

- Can you tell me more about that?
- Can you tell me more about how that affects [X]
- Can you clarify what you mean by [significant statement]
- Can you give me an example of a time when [significant statement] happened to you

**Conclusion**

Is there anything else you want us to know about [X]? (YES return to interview; NO proceed)

We want to thank you so much for your participation and remind you that everything we have discussed will remain private. The audio file will be destroyed once this interview is transcribed, and the transcription will not contain your name or any identifying information.

**Interview Guide 2: Patients Living with HIV**

**Training Note:** Not all questions will be asked explicitly. Participants may answer the questions, or cover the topic, in the course of the focus group or one-on-one conversation.

**Introduction**

[Remind about Audio Recording and not to use real names]

Thank you for talking with us today. We are interested in learning more about your thoughts and beliefs about cardiovascular or heart disease. I am going to ask you some questions about these topics to hear about your experiences and perspectives. Please know that there is no right or wrong answer. You will notice that I won’t give you a lot of feedback on your responses because I don’t want to influence your answers. You may also notice that I will jot things down on paper while you talk- this is simply a reminder to ask you a follow-up question. You are under no obligation to talk about anything that you are not comfortable discussing with me. But we do want everyone to have a chance to speak. It’s important that we hear from everyone throughout the conversations. Finally, we’d like everything and everyone to be kept confidential. Please do not repeat anything you hear in the focus group outside of this room. Do you have any questions or concerns before we begin?

[Facilitator, please note in your reflections the number of participants present, their gender breakdown and the start and stop time of the interview.]

**Primary Questions**

***General CVD Perceptions***

1. Let’s start by asking you to tell me about what you know about cardiovascular or heart disease. **Prompts**: What is heart disease; what causes heart disease; how do you prevent it; how do you treat it if you get it; how do you know if you have it?
2. Do you think you are at risk for developing heart disease? Why? **Prompts**: Family history, age, stress, past or current substance use, obesity, inactivity, diet, medications you take, tobacco use, HIV medication you take,
3. What is a stroke? What do you know about the risk factors for stroke? Probe: what similarities or differences are there between stroke and other heart disease risk factors
4. Has your doctor or nurse has talked with you about your risk for having a heart attack or stroke? Tell me about those discussions. **Prompts**: What prompted that discussion (e.g., did you bring it up, was it part of a research study, did you have abnormal blood pressure or cholesterol?). What evaluations were done and what action plan was discussed or agreed upon?
5. Do you have a separate primary care doctor (from your HIV doctor)? If no, skip to 5. If yes, why do you choose to have this separate provider? Probe? What aspects of risk for heart disease do you talk about? How does communication flow between your HIV doctor and your PC doctor?
6. Which medications do you use to prevent heart attacks and strokes?
7. What are some of your concerns about taking medications to prevent heart attacks or strokes? **Prompts**: side-effects, being worried about what will happen if you don’t take the medications, costs, access? What tradeoffs do you make with these concerns? Probe: not taking medications? Take them despite concerns, reduce frequency of taking them?
8. Are you adherent taking your HIV meds? Are you or do you think you would be as adherent taking heart/stroke meds? Why or why not?
9. Tell me about your practice of monitoring your blood pressure. Probe: apart from the doctor’s office, where else do you check your blood pressure? Why do you choose this/ these locations and how frequently do you do it?
10. What else do you do to help prevent heart problems? Who or what helps you do that?

***HIV and CVD***

Next we’re going to talk about both HIV and heart disease.

1. Do you think HIV affects your risk for heart disease? If so, how?
2. Has anyone in your doctor’s office ever talked with you about the relationship between HIV and heart disease? Who? What did they tell you? How did you respond to that conversation? Did it impact how you take care of yourself? How?
3. If you knew that HIV and HIV medication increases your heart disease risk, how would this impact your behavior? (e.g., smoking, diet, exercise, taking other non-HIV medications, seeing/talking with your doctor).
4. What concerns do you have with other medications interacting with your HIV drugs? If a non-HIV doctor prescribes a medication for you, do you always check with your HIV doctor before you agree to take it? Do you rely on your pharmacy to check for possible interactions with your other medications?
5. Would you be more likely to take a preventive medication (like aspirin or statins) if your HIV doctor prescribed it compared to a non-HIV provider? Why or why not? Who else influences your decision to take medications for your heart?

***Intervention Tailoring***

1. Tell me about a time you improved your health behavior. For example, a time when you quit smoking or lost weight. What helped you achieve this improvement? What steps did you take to make the change? Has this been a permanent change? If not, how long did it last? What challenges did you face on the way?
2. Do you ever track your health conditions at home, like your weight, blood pressure, sugar levels or your mood? How do you do it? **Probe**: keep logs? What would make it easier for you to do so? What if we gave you a kit to monitor your blood pressure? How would your frequency of checking be affected? What concerns would you have? What would make it easier?
3. Other than your doctor, where do you get health information? What sources do you trust? Do you get information about your health from other patients with HIV (e.g., support groups)?
4. What sorts of health messages or education do you prefer? Find motivation?
5. Thinking about your lifestyle and routines, what sort of intervention or program might help you improve heart disease prevention behaviors such as smoking cessation, eating a healthier diet, talking with your doctor about how you can determine your risk for heart disease?
6. Do you prefer paper, verbal (conversations), digital (phone, computer) or visual (TV) messages to help you understand health information the best and how to best act on that information to improve your health?

**Secondary/Follow-up Questions**

Follow-up “Probes” after significant statements are made:

[Earlier/A moment ago/when you first started speaking/when you were talking about x] you said [significant statement].

- Can you tell me more about that?
- Can you tell me more about how that affects [X]
- Can you clarify what you mean by [significant statement]
- Can you give me an example of a time when [significant statement] happened to you

**Conclusion**

Is there anything else you want us to know about [X]? (YES return to interview; NO proceed)

We want to thank you so much for your participation and remind you that everything we have discussed will remain private. The audio file will be destroyed once this interview is transcribed, and the transcription will not contain your name or any identifying information.

**Interview Guide 3: Nurses, Nurses Assistants, Social Workers, Case Managers & Dieticians**

**Training Note:** Not all questions will be asked explicitly. Participants may answer the questions, or cover the topic, in the course of the focus group or one-on-one conversation.

**Introduction**

[Remind about Audio Recording and not to use real names]

Thank you for talking with us today. We are interested in learning more about your thoughts and beliefs about cardiovascular disease among your HIV+ patients. We are most interested in the patients you see in their routine, outpatient HIV care, not those in the hospital. I am going to ask you some questions about these topics to hear about your experiences and perspectives. Please know that there is no right or wrong answer. You will notice that I won’t give you a lot of feedback on your responses because I don’t want to influence your answers. You may also notice that I will jot things down on paper while you talk- this is simply a reminder to ask you a follow-up question. Finally, you are under no obligation to talk about anything that you are not comfortable discussing with me. Do you have any questions or concerns before we begin?

[Facilitator, please note the participant’s ID number in the audio recording so we can match it to the demographic information.]

***General CVD Perceptions***

1. Let’s start by you telling me about what you think the biggest health problems are for your HIV+ patients. For those health problems, how do think they compare with the general HIV + population in the US?
2. Can you tell me what you know about cardiovascular or heart disease and HIV.
3. Have you ever talked with your patients about heart disease? If yes, tell me about that/those conversation(s)?

**Prompts**: What prompted that discussion (e.g., did you bring it up, was it part of a research study, did you have abnormal blood pressure or cholesterol?) Did they ask you about their risk of developing heart disease? Did their primary care provider reach out to you?

1. What medications are used to prevent heart attacks and strokes?
2. What can do you do to help prevent heart problems in your HIV+ patients? Who or what helps you do that?

***HIV and CVD***

1. What have you ever heard about HIV medications affecting your patient’s risk of developing heart disease?
2. How does knowing they are at increased risk for heart disease affect your patients’ health behavior (e.g., smoking, diet, exercise, taking medications, seeing/talking with your doctor).
3. What interventions does your clinical current use to improve the heart health of your patients? Tell me about them? Are there other interventions to improve the heart health that might work in your clinic? How would you design them?

**Nurse and Medical Assistant ONLY questions**

- Can you tell me when in the patient visit your patients’ blood pressure is assessed? What are the steps involved in measuring it? When you think about the process of taking blood pressure measurement, what makes it easier or harder to achieve fidelity in the standard process? Probe: Is it logistically difficult for the patient to rest quietly for a time before it is taken?
- How often do you reconcile your patients’ medications? Do they ever have questions about their blood pressure and cholesterol medications? What sorts of questions do they ask? How do you address the questions?
- Do you assess medication adherence? For which medications? Tell me about the feedback you provide based on the medication adherence levels? What do you do if they are non-adherent? If they are non-adherent to any of their medications do you follow up with them after their visit to see if its changed? Do these strategies include medication adherence for cardiovascular medications? Why or why not?

***Intervention Feasibility and Usability***

Finally, we are interested in designing a new initiative, with a new nurse coordinator, to help your patients reduce their risk of heart disease. However, we know in this and many clinics, there are cultural and systemic norms that can make a new clinic initiative more or less successful. We’d like to get your opinion on these issues and as a reminder, all information you provide will be confidential and aggregated across three clinics.

*System*

1. Tell me about your clinic’s culture. What aspects of it help you do your job well and what aspects can make it harder for you? What are the strengths of your clinic? The weaknesses?
2. Does your clinic have a hierarchy? If so, can you tell me how that affects how decisions are made in your clinic?
3. What the priorities or the mission of your clinic? Have they changed over time?
4. Tell me about a clinic initiative that was recently developed to help your patients improve their health behavior. What steps were taken to help make the change? Who initiated the change and how did having that person champion it affect the initiative? How long did it last? What was the outcome (did it work)? How were individuals or groups supported as they adjusted to the new changes? During implementation, how were problems solved? What indicators were there that people were interested and committed in implementing the changes brought by the initiative?
5. What factors do you think would encourage clinicians to adopt a new program to improve the heart health of patients in your clinic?

*Training*

1. What sort of education or training do you prefer for new initiatives? Do you prefer paper, verbal (conversations), digital (phone, computer) or visual (TV) training? Individual or Group? Why? What are your thoughts about continuing education credit? Probe: entice you to complete new trainings? Would you be interested in helping to develop a new training for your clinic on reducing heart disease in your patients?

*Design Considerations/Representations*

1. When you have a patient concern, for example an elevated temperature or a high blood pressure, how does that information get turned into action in this clinic? Can you walk me through the various steps? [consider drawing this out on white board]. How do you involve non-HIV clinicians, for example your patient’s primary care provider, in this communication cycle?

2.How often do you personally communicate with your patients outside of the clinic setting? How does this communication happen (e.g., phone, EMR). Does the patient usually initiate this contact or do you?

**Secondary/Follow-up Questions**

Follow-up “Probes” after significant statements are made:

[Earlier/A moment ago/when you first started speaking/when you were talking about x] you said [significant statement].

- Can you tell me more about that?
- Can you tell me more about how that affects [X]
- Can you clarify what you mean by [significant statement]
- Can you give me an example of a time when [significant statement] happened to you

**Conclusion**

Is there anything else you want us to know about [X]? (YES return to interview; NO proceed)

We want to thank you so much for your participation and remind you that everything we have discussed will remain private. The audio file will be destroyed once this interview is transcribed, and the transcription will not contain your name or any identifying information.
